# Machine Learning Suggests That Small Size Helps Broaden Plasmid Host Range

**DOI:** 10.3390/genes14112044

**Published:** 2023-11-05

**Authors:** Bing Wang, Mark Finazzo, Irina Artsimovitch

**Affiliations:** Department of Microbiology and Center for RNA Biology, The Ohio State University, Columbus, OH 43210, USA; finazzo.2@buckeyemail.osu.edu

**Keywords:** conjugation, horizontal gene transfer, plasmid host range, small-size plasmid, antibiotic resistance genes, clustering, machine learning

## Abstract

Plasmids mediate gene exchange across taxonomic barriers through conjugation, shaping bacterial evolution for billions of years. While plasmid mobility can be harnessed for genetic engineering and drug-delivery applications, rapid plasmid-mediated spread of resistance genes has rendered most clinical antibiotics useless. To solve this urgent and growing problem, we must understand how plasmids spread across bacterial communities. Here, we applied machine-learning models to identify features that are important for extending the plasmid host range. We assembled an up-to-date dataset of more than thirty thousand bacterial plasmids, separated them into 1125 clusters, and assigned each cluster a distribution possibility score, taking into account the host distribution of each taxonomic rank and the sampling bias of the existing sequencing data. Using this score and an optimized plasmid feature pool, we built a model stack consisting of DecisionTreeRegressor, EvoTreeRegressor, and LGBMRegressor as base models and LinearRegressor as a meta-learner. Our mathematical modeling revealed that sequence brevity is the most important determinant for plasmid spread, followed by P-loop NTPases, mobility factors, and β-lactamases. Ours and other recent results suggest that small plasmids may broaden their range by evading host defenses and using alternative modes of transfer instead of autonomous conjugation.

## 1. Introduction

Since 1952, when Joshua Lederberg proposed the term plasmid for any extrachromosomal genetic material [1], plasmid replication, mobility, and maintenance have been extensively studied [2]. While the role of plasmids in shaping bacterial evolution and their utility as investigative and biotechnological tools are broadly recognized by the scientific community, their contribution to the rapid spread of antibiotic-resistant genes (ARGs) has made plasmids a pressing medical threat. The existence of ARGs predates the modern era of antibiotic use [3], but conjugative plasmids isolated from infected humans prior to 1954 rarely carry ARGs [4]. The widespread, and often reckless, use of antibiotics in medicinal practice, and even more so in farming [5], exerted strong selective pressure for ARG acquisition by human- and livestock-associated bacteria, and plasmids carrying multiple ARGs are common in samples sequenced today [6].

Plasmids are ideal vehicles for disseminating ARGs by horizontal transfer [7]. Plasmid transfer can occur across phyla in soil [8] and in the gut [9], and even across domains [10]. The omnipresence of ARGs in commensal microbiomes [11] renders even the last-resort antibiotics ineffective in the treatment of infectious diseases [12] and compels us to understand the mechanisms that enable their spread.

Plasmids deliver their cargo into new hosts through conjugation and are classified into three types based on their mobility: conjugative, mobilizable, and non-mobilizable plasmids [7]. Conjugative plasmids encode the origin of transfer (oriT), relaxase (MOB), and membrane-associated mating pair formation (MPF) complex, the elements required for autonomous transfer. Mobilizable plasmids do not carry the complete set of transfer elements but can hijack the missing factors from co-resident conjugative plasmids, and non-mobilizable plasmids lack components that mediate transfer.

While efficient conjugation is a prerequisite for plasmid spread, plasmids contain numerous other genes that determine their success. A newly acquired plasmid must evade cellular defenses, such as restriction-modification (RM) systems. To persist in a new host, plasmids must efficiently replicate and be partitioned between daughter cells. Novel biosynthetic pathways and resistance to antibiotics and heavy metals promote survival under selective pressure [13]. Other factors may neutralize host-encoded defense systems [14] or act as addiction modules that prevent plasmid loss [15]. The current understanding of plasmid persistence was extensively reviewed, and a central question was posed [16]: what drives the distribution of plasmids in bacterial (pan)genomes?

Clustering analysis of plasmid homologs can be used to analyze their host range and identify factors that determine this range. Several classification approaches have been developed; the most sophisticated methods are based on the partitioning of networks inferred from genetic distances between entire plasmid sequences [17]. Many tools for the prediction of the plasmid host range have been published. MOB-suite is based on the types of replicons, oriT, MOB, and MPF [18]. COPLA works by assigning plasmid taxonomic units (PTUs) based on total average nucleotide identity (ANI) [19]. PlasmidFinder uses the BLASTn algorithm to look for DNA homologies [20]. PLACNET, which builds a contig interaction network using reference plasmids from different hosts, can infer potential plasmid–host associations directly from whole genome sequencing data [21]. The rapidly increasing plasmid sequence space, combined with the enormous diversity of plasmid-encoded genes, pose challenges for conventional bioinformatics approaches and call for the application of machine-learning algorithms to harness this wealth of data [22]. For example, PlasmidHostFinder, a trained random-forest model, shows advantages over PlasmidFinder on incomplete plasmid sequences [23]. The newly released HOTSPOT [24] incorporates a language model, Transformer, and takes all predicted proteins into account, outperforming MOB-typer [18], PlasmidHostFinder [23], and PlasFlow [25].

Here, we resolved plasmid clusters for a complete dataset of 30 thousand bacterial plasmids from NCBI by using the Leiden algorithm [26]. Plasmid backbone features including size, topology, and GC content, and the gene function pool of the plasmidome, i.e., all plasmids collectively, were fed into machine-learning models to identify the features that determine host ranges. Our results demonstrate that P-loop NTPases, involved in a wide range of cellular processes including DNA and protein translocation, and metallo-β-lactamases, whose abundance reflects the wide use of β-lactam antibiotics, have impacts on plasmid spreading. However, our analysis identifies a small size (under 20 kbp) as the most important feature for model stack performance.

## 2. Materials and Methods

### 2.1. Plasmid Sequences Collection

The plasmid list was downloaded from NCBI (https://ncbi.nlm.nih.gov/genome/browse#!/plasmids/, accessed on 17 April 2023 ). The sequence completeness was checked with EDirect (v13.7) [27]: if a plasmid belonged to an assembly, a “complete genome” level is required; otherwise, the completeness status record must be “complete”. Then, all of the complete bacterial plasmid sequences were downloaded from NCBI. Finally, all sequences were passed through Deeplasmid [28] to check for potential bacterial chromosomes. Only plasmids that can be reliably processed by Deeplasmid (1–330 kbp) and labeled as “plasmid” were kept in our dataset, whereas ones labeled as “genome” were removed. Sequences labeled as “AMBIGUOUS” were compared with PLSDB (2021-6-23 V2) [29], and only those that were in the PLSDB were kept. To reduce the redundancy, the whole plasmid sequence was sketched (options: −s 330000 −k 21) and the mash distance was calculated using Mash (v2.3) [30]. Plasmids that have 100% identity (mash distance = 0) and come from the same species were grouped, and one representative was randomly selected from each group.

### 2.2. Plasmid Clustering

The pairwise ANI values among plasmids were calculated using FastANI (v1.33) [31]. The query plasmid was fragmented into 200 bp fragments and 50% of the smaller plasmid had to be orthologous to the larger plasmid for trusting ANI (options: --fragLen 200 --minFraction 0.5). Self-comparison was removed, and reciprocal pairs were merged by averaging the ANI values. A transformed ANI has been successfully used for clustering plasmids [32,33]. Although 95% ANI is argued not to be a universal threshold for the species boundary [34], two sequences with ≥95% ANI values will certainly be very similar. Thus, we transformed the ANI values into edge weights as follows:

(1) $$\mathrm{edgeweights}=\left\{\begin{array}{c} \begin{array}{c} \;\;\;\;\;\;\;\frac{ANI}{100}\;;\mathrm{if}\;ANI\;\geq 95\\ \frac{1}{1+20\left(1-\frac{ANI}{100}\right)}\;;\mathrm{otherwise} \end{array}\;\;\;\;\;\;\;\; \end{array}\right.$$

In Equation (1), *ANI* indicates the ANI values.

Plasmids clustering was performed by using the Leiden algorithm with the constant Potts model [26] implemented in Python (v3.10.4). To find the best partitions, we performed a resolution scan (ranging from 0 to 1). The high modularity value here indicates dense connections inside of modules but loose connections among nodes of different modules. Groups with more than two members were kept for further analysis (Dataset 1A), whereas others were removed. All the removed plasmids are listed in Dataset 1B. All networks were visualized using Cytoscape (v3.9.1).

### 2.3. Distribution Possibilities

For each cluster, we calculated a distribution possibility based on the taxonomic lineage of bacterial hosts (Figure S1). (1) For a given cluster, the number of plasmids of each taxon (Pn) was calculated for taxonomic rank genus, family, order, class, and phylum (Dataset 1C). (2) Since our dataset is biased (Figure S2, many taxa will be underrepresented), and the plasmid number of each taxon had to be normalized. For a given taxon, which is present in several clusters, the average plasmid number per cluster (*APn_tax_*) can be calculated. *Pn* was then normalized as follows:

(2) $$\left\{\begin{array}{c} {NPn}_{tax}=1;\mathrm{if}\;Pn\geq {APn}_{tax}\\ {NPn}_{tax}=\frac{Pn}{{APn}_{tax}};\mathrm{otherwise} \end{array}\right.$$

where *NPn_tax_* is the normalized plasmid number for a given taxon.

(3) Based on *NPn_tax_*, we first calculated a standard deviation (*SD_rank_*) for a given taxonomic rank using Equation (3) to capture the plasmid distribution. A smaller *SD_rank_* suggests that the plasmids are more evenly distributed across the taxa for a given rank:

(3) $$\left\{\begin{array}{c} {SD}_{rank}=\sqrt{\frac{\sum_{i=1}^{x}\left({NPn}_{tax\_i}-{\left.{APn}_{rank}\right)}^{2}\right.}{x}};\mathrm{if}\;x>1\\ {SD}_{rank}=1;\mathrm{otherwise} \end{array}\right.$$

where *x* is the number of taxa for a given rank, and APn_rank_ is the average of *NPn_tax_* for a given rank. Then, the plasmid distribution score (*DS_rank_*) is calculated as follows:

(4) $${DS}_{rank}=\left(1-{SD}_{rank}\right)x\;\;\;\;\;\;$$

Thus, for a given rank, the more taxa it has, the bigger is the *DS_rank_*.

(4) The *DS_rank_* is converted into the distribution possibility (*P_dis_rank_*) (Dataset 1C) as follows:

(5) $$\left\{\begin{array}{c} P_{dis\_rank}=1;\mathrm{if}\;{DS}_{rank}>2\\ P_{dis\_rank}=\frac{{DS}_{rank}}{2};\mathrm{otherwise} \end{array}\right.\;\;\;\;$$

*P_dis_rank_* describes the possibility that a cluster can spread on a given rank. For example, if the rank has two taxa and *SD_rank_* = 0 (a perfectly even distribution), then *DS_rank_* = (1 − 0) × 2 = 2 according to Equation (4), and this cluster was defined to have 100% possibility to spread on this rank; see Equation (5).

Finally, a sum of the distribution possibility (*P_sum_*) (Dataset 1C) is calculated for each cluster as follows:

(6) $$P_{sum}=\sum P_{dis\_rank};rank=\mathrm{genus},\;\mathrm{family},\;\mathrm{order},\;\mathrm{class},\;\mathrm{phylum}$$

### 2.4. Extraction of Features

Plasmid size, GC content, and topology were extracted from NCBI. MOB-typer (v3.1.4) [18] was used to identify types of replicon(s), MOB, MPF, and oriT. All plasmids were annotated using prokka (v1.13) [35] with evalue < 1 × 10^−9^. The predicted proteins were searched against Pfam_A models (https://www.ebi.ac.uk/interpro/download/pfam/, accessed on 25 June 2023) using hmmscan (v3.2.1) (http://hmmer.org/) with model-specific trusted cutoffs. The COG terms [36] were assigned using the cdd2cog script (v0.1) [37]. To identify putative ARGs, the predicted proteins were analyzed using AMRFinder (v3.11.18) [38] and blastp searching against the CARD database [39] (evalue < 1 × 10^−10^, bitscore > 50).

To prepare for machine-learning model training, the collected features were processed through multiple steps. (1) The plasmid sizes were converted into categorical values as follows: smallest (<20 kbp), small (20–50 kbp), average (50–100 kbp), and large (>100 kbp). GC (%) content follows the rule: lower (<30), low (30–40), mean (40–50), high (50–60), and higher (>60). The features collected through MOB-typer and hmmscan are categorical values already. (2) The fractions of all categorical values were calculated for each cluster. (3) All features were combined and less informative clusters, with fewer than 22 features (~2% of the ones with the richest features), were removed. (4) All *P_sum_* = 0 were replaced with *P_sum_* = 0.3, the lowest value besides *P_sum_* = 0. Although both values indicate that the cluster is limited at the species level, *P_sum_* = 0.3 improves model performance. (5) All features were scaled to 0–1, calculated column-wise using MinMaxScaler from ScikitLearn (v0.7.0) implemented in Julia (v1.9.1). (6) Features with less than 1% variation calculated column-wise were deleted from the data matrix. (7) Using the features from step 6, a model stack was trained as described below. Then, the feature columns were eliminated one by one and the feature that had the greatest negative effect on the model stack performance was removed. A total of four rounds were performed. The final data matrix (920 × 141) contained 920 observations (clusters), each having one *P_sum_* value and 140 features.

### 2.5. Model Training

The model training was performed with MLJ (v0.19.2) [40]. First, six base models (CatBoostRegressor, EvoTreeRegressor, RandomForestRegressor, DecisionTreeRegressor, LGBMRegressor, and XGBoostRegressor) were optimized separately. The data matrix was split into training and testing datasets randomly at a ratio of 4:1. The models were tuned on the training dataset and the performance was evaluated on the unseen testing dataset. A Cartesian grid-based hyperparameter tuning strategy with default resolution and five-fold cross-validation was deployed. Because ~85% of the *P_sum_* were <1.2 (Dataset 1C), we applied the synthetic minority over-sampling technique (SMOTE) [41] to the training dataset to triple the data points with *P_sum_* = 1.2–2.5 and quadruple those with *P_sum_* = 2.5–5.

A model stack might outperform all the base models [42]. After tuning the base models, LinearRegressor was used as a meta-learner for searching the best combinations of the base models, as it proved to be superior to other models, such as EpsilonSVR, LassoRegressor, and RidgeRegressor.

### 2.6. Importance Index

To assess contributions of individual features to the model stack performance, we used the drop-column importance method: (1) The feature columns were removed one by one to generate updated data matrices. (2) The updated data matrices were split into training (80%) and testing (20%) datasets randomly. (3) The model stack was trained on the training dataset and tested on the unseen testing dataset. (4) Since different splits cause different model performance, steps 1–3 were repeated 100 times to obtain the mean of the mean squared error (MSE). Stack model performance on the original data matrix serves as a baseline, allowing us to use changes of MSE to indicate the feature importance. Finally, the MSE differences were normalized by the greatest changes. The normalized values were used as the importance index (Dataset 1D).

### 2.7. Feature Combinations

Next, we explored the combined effects of selected features. Our data matrix had 140 features, and it would be impossible to test all possible feature combinations, ~10^42^. To identify the most impactful feature combinations, we first tried to drop two feature columns at the same time; however, none showed greater effect than removing only “smallest”. Then, we launched a “lucky guess” to find the best combination that could significantly compromise the model stack performance: (1) The data matrix was split into training (80%) and testing (20%) datasets randomly. (2) A combination consisting of *n* (*n* = 4, 5, 6, 7, 8) features was generated randomly. (3) Since “smallest” has the highest importance index (Dataset 1D), the selected features from step 2 and “smallest” were dropped to generate an updated training and testing dataset. (4) The model stack was trained on the updated training dataset and tested on the testing dataset to obtain the MSE. If the MSE was greater than the initial standard, the initial standard was updated to the MSE and the feature combination was recorded. (5) We repeated steps 2–4 50,000 times.

## 3. Results

### 3.1. Defining Features of Bacterial Plasmids

Many plasmid databases have been compiled for different purposes, e.g., COMPASS [43], PLSDB [44], and Plasmid MLST [45]. However, these databases are either outdated (an issue compounded by the rapid expansion of the plasmidome) or have only highly selected representatives. In our work, we used a complete bacterial plasmid dataset from NCBI composed of 30,464 plasmids.

**Figure 1:**
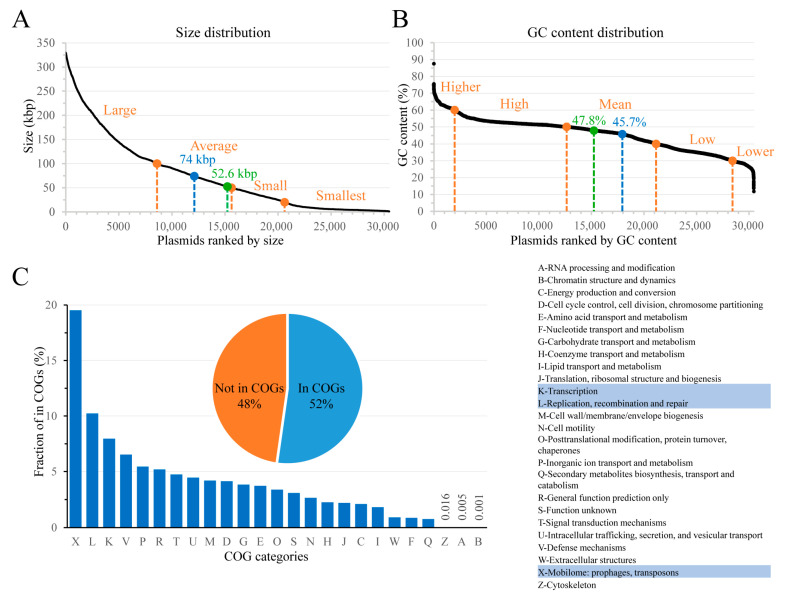
Overview of the dataset. Plasmid size (**A**) and GC content (**B**) distribution. Mean and median values are indicated by blue and green dots, respectively. The cutoffs for categorical terms are indicated with orange dots. (**C**) Distribution of plasmid-borne proteins among COGs (shown on the right). The pie chart shows the proportion of proteins in COGs. The bar chart shows the fraction of proteins assigned to a given COG; fractions of the Z, A, and B categories are indicated by numbers. The three most prevalent COG categories are highlighted in the table.

Each plasmid is characterized by a unique combination of structural and functional features that could shape its host range. The structural features include plasmid topology (linear vs. circular), GC content, and size (length of nucleotide sequence), and can be easily defined. The collected plasmids have an average size of 74 kbp and an average GC content of 45.7%. The median values for size and GC content are 52.6 kbp and 47.8%, respectively (Figure 1A,B). Plasmid-encoded proteins represent functional features that are much more difficult to define. Our dataset contains 2,539,260 predicted proteins whose functions can be hypothetically assigned based on similarity to known proteins. The Clusters of Orthologous Groups (COGs) database allows classification of proteins into very broad functional groups based on their phylogenetic relationships [46]. We assigned COG categories to all predicted proteins in our dataset (Figure 1C) to find that plasmid-borne proteins appear to have very diverse functions, falling into 25 out of 26 COG categories; the A, B, and Z COG categories are underrepresented, and no proteins belong to the Y (nuclear structure) category. As expected, the top three categories, comprising ~37% of proteins that are in the COGs, represent functions related to plasmid replication and propagation (Figure 1C). Notably, only half of the plasmid-encoded proteins can be assigned to COGs, thus representing conserved protein families, whereas the remaining predicted proteins likely have novel functions [46].

The immense diversity of plasmid genomes makes elucidating the relationships between all plasmid features and their host range challenging for traditional bioinformatic methods. Here, we explored machine learning-based models to identify the key features that determine plasmid spread. However, using too many features would introduce noise and slow down computing. To reduce the number of features, plasmid size and GC content values were quantized into arbitrary sets, and predicted proteins were grouped into protein families/domains.

Based on the study of plasmid mobility types versus plasmid sizes [7] and commonly used size cutoffs, we divided plasmids into four categories by size, each containing a similar number of plasmids (Figure 1A). The GC content changes roughly linearly in our dataset except for the extreme values (>~60% or <~28%), which were assigned to the “higher” and “lower” categories, respectively. The remaining plasmids were separated into three categories with 10% GC intervals (Figure 1B). Conserved protein function classification was performed using Pfam-A models [47]. A total of 7085 unique Pfam models were assigned to the predicted proteins. The classification of conjugation machinery components has been used for plasmid host prediction by Mob-typer [48]. We assigned 745 replicon types, 10 MOB types, 5 MPF types, and 6 oriT types to all plasmids. The ribosomal RNA, tRNA, ncRNA, and DNA/RNA regulatory elements were not considered.

An initial set of 7862 features did not support good model performance, prompting us to reduce the number of features to minimize noise. After several steps of feature engineering, the pool displaying the best performance contained 140 features (Dataset 1D).

### 3.2. Plasmid Clustering

**Figure 2:**
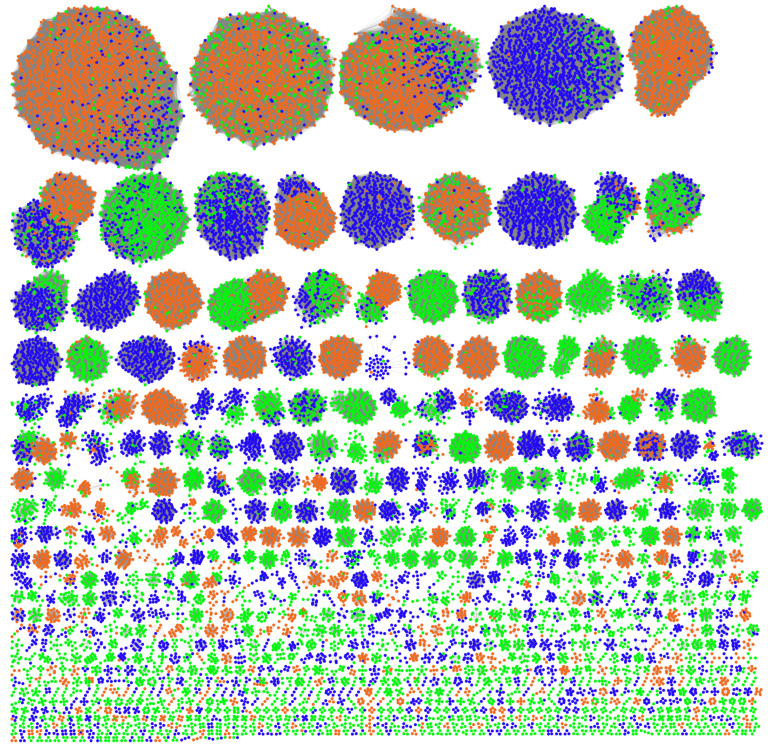
Plasmid clustering. Every node represents a plasmid. The network includes 30,464 plasmids assigned into 1125 clusters (≥3 members). The nodes are colored according to the three plasmid mobility types: Orange, conjugative. Blue, mobilizable. Green, non-mobilizable.

To define their host distribution, we clustered all the plasmids first. The pairwise ANI has been successfully used for plasmid clustering [32,33]. In this study, we used transformed ANI to build a plasmid similarity network and identified 1125 clusters (Figure 2) using the Leiden algorithm with the constant Potts model [26]. The final network achieved a modularity of 0.8 with a resolution of 0.02 (Figure S3); a high modularity value, which ranges from 0 to 1, implies good cluster separation. The three mobility types are nearly equally represented in our dataset (Dataset 1A) and plasmids of different mobility types co-exist in many clusters, but one mobility type is predominant in most (Figure 2). Non-mobilizable plasmids tend to be found in small clusters, reflecting limitations on genetic information exchange with other bacteria.

### 3.3. Plasmid Distribution

**Figure 3:**
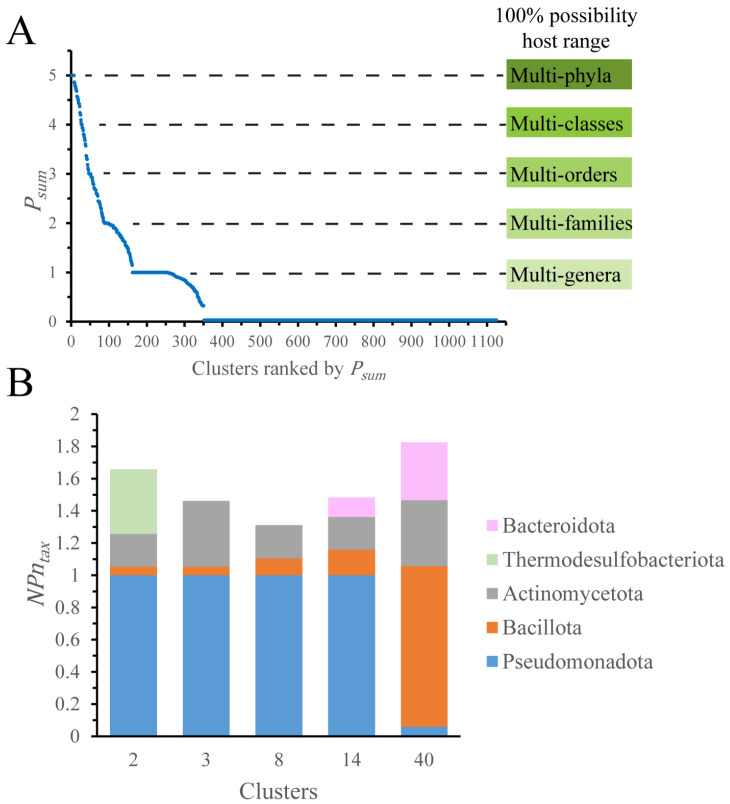
Defining the plasmid distribution possibility. (**A**) The sum of distribution possibility (*P_sum_*). Meanings of the *P_sum_* for distribution levels are indicated. (**B**) Phyla distribution for clusters with *P_sum_* > 4.7. Only clusters with more than 100 plasmids were considered. *NPn_tax_*: for a given taxon, the plasmid number is normalized by the average plasmid number per cluster.

We chose to study plasmid distribution based on the collective behavior of an entire cluster, rather than on individual plasmids. To describe the host distribution in each cluster, we used a sum of distribution possibility scores (*P_sum_*). The *P_sum_* ranges from 0 to 5, with 5 indicating 100% possibility for multi-phyla distribution (Figure 3A, Dataset 1C).

Our analysis shows that only 13 out of 1125 clusters have more than 70% possibility (*P_sum_* > 4.7) for multi-phyla distribution, whereas 805 clusters have *P_sum_* < 0.7, indicating 70% possibility of species-level distribution. The majority of hosts in multi-phyla clusters are limited to one phylum, Pseudomonadota or Bacillota (Dataset 1C, Figure 3B). For instance, cluster 2 contains 1266 plasmids of Pseudomonadota and one each of Actinomycetota, Bacillota, and Thermodesulfobacteriota. The sequencing bias is one reason responsible for this distribution pattern. Plasmids from Pseudomonadota and Bacillota comprise 93% of our dataset, and the Enterobacteriaceae family accounts for almost three-quarters of plasmids from Pseudomonadota (Figure S2). This bias complicates the analysis of plasmid distribution on the levels higher than a family.

To mitigate this bias, the plasmid number of a taxon was normalized (*NPn_tax_*) by the average plasmid number per cluster (*APn_tax_*) for the *P_sum_* calculation. After normalization, the differences among phyla became much smaller, yet Pseudomonadota and Bacillota remained predominant in multi-phyla clusters (Figure 3B).

### 3.4. Machine-Learning Models Training

**Figure 4:**
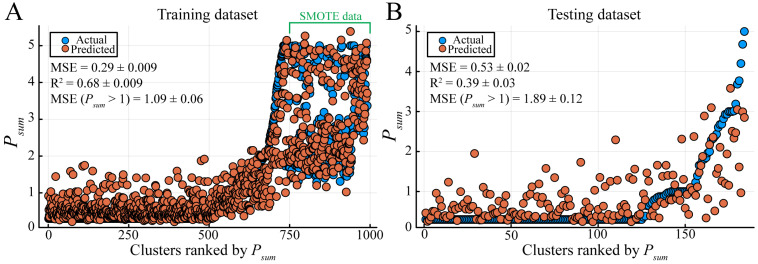
Performance of the final model stack. The dataset was split 100 times randomly into training (**A**) and testing (**B**) dataset pairs. The model stack was trained on the training dataset, and then tested on paired unseen testing datasets. MSE, mean squared error. R2, coefficient of determination. MSE (*P_sum_* > 1), MSE for those clusters with *P_sum_* > 1. Values are reported as mean ± SD (*n* = 100). The data points on the very right of the training dataset were synthesized by SMOTE.

Plasmid-encoded ARGs confer an obvious selective advantage in agricultural and clinical settings and are thus often discussed in light of their alarming spread through bacterial communities, whereas metabolic genes affect conjugation and antibiotic susceptibility indirectly [49]. How significant is the ARGs’ influence on plasmid spread? In our dataset, the number of ARGs-containing plasmids is not correlated with *P_sum_* (Figure S4A, Dataset 1E) and more features need to be considered to comprehensively analyze their effects on the plasmid distribution. Using the calculated *P_sum_* and defined features, we explored tree-based and gradient-boosting algorithms, which show advantage over deep learning models on a small-size dataset organized in a tabular style [50,51]. The base models were trained and evaluated separately (Table 1, Figure S5). Then, a model stack was used to optimize performance (Table 1). After testing all combinations, three base models, namely DecisionTreeRegressor, EvoTreeRegressor, and LGBMRegressor, were stacked with LinearRegressor as the meta-learner. The model stack outperformed all base models except for LGBMRegressor, as indicated by the MSE (Figure 4 and Figure S5), and worked better than LGBMRegressor for clusters with *P_sum_* > 1 (Figure 4 and Figure S5). However, the prediction of model stack for the clusters with *P_sum_* > 3 was poor (Figure 4), which is not surprising since we do not have many clusters showing distribution above order levels (Figure 3A, Dataset 1C). We also saw large fluctuations for several data points with *P_sum_* = 0, which may be a result of not having enough sequencing data to classify these clusters correctly.

### 3.5. Feature Importance Analysis

**Figure 5:**
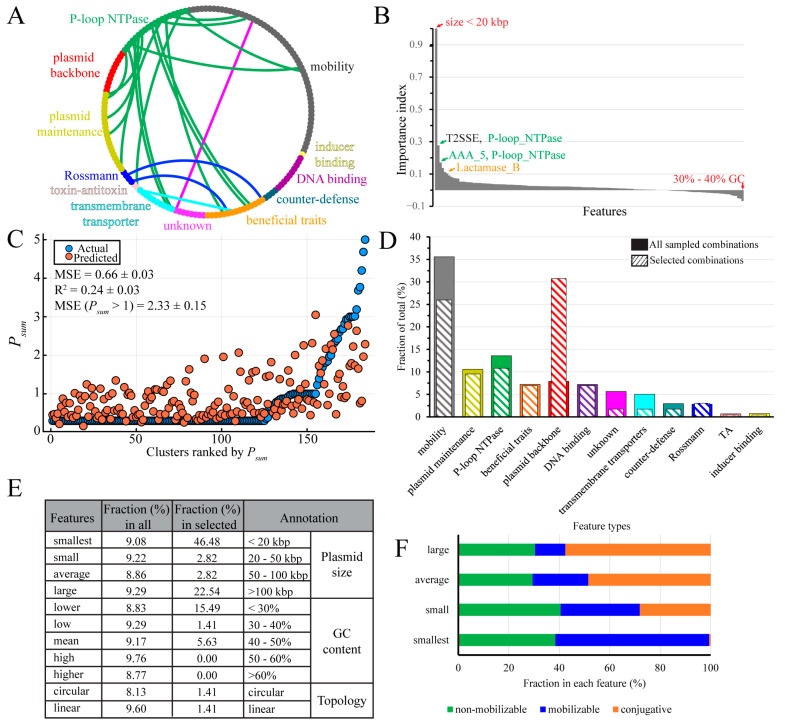
Feature importance analysis. (**A**) An overview of feature types. The outer rim of the diagram represents features grouped into 12 main feature types. The mobility of feature types are features involved in plasmid mobility, which is not the same as the three plasmid mobility types. Features that co-occur in different types are linked by edges. (**B**) Importance index calculated by drop-column analysis. Positive values indicate that the feature removal worsens model stack performance, whereas negative values indicate features whose removal improves model performance. An importance index of 0 indicates that feature removal has no effect. (**C**) Model stack performance on testing dataset after removal of “smallest”. Values are reported as mean ± SD (*n* = 100). MSE was calculated by using 100 randomly split training and testing dataset pairs as in Figure 4. (**D**) Feature type distribution analysis from randomly sampled seven-feature combinations. All sampled combinations, the 11,000 combinations. Selected combinations, the 33 selected combinations. (**E**) Detailed analysis of the backbone features in all vs. selected combinations. (**F**) The mobility types distribution in plasmids of different sizes.

Because the model stack fails to understand clusters with *P_sum_* > 3, this analysis is only expected to reveal features important for distribution below the order level. All features were grouped into 12 types (Figure 5A; see Dataset 1D for members of each type). Next, we used drop-column importance analysis, where the model’s performance is tested after the removal of individual features, to calculate the importance index for each feature (Figure 5B, Dataset 1D).

Most features had a positive importance index, with the “smallest” size (<20 kbp) having the greatest effect on the model stack performance; a weak correlation was observed between *P_sum_* and “smallest”, but not with other plasmid sizes (Figure S4B–E). P-loop NTPases comprised the second most important group of positive features and ARGs also stood out, with the metallo-β-lactamase superfamily (Lactamase_B) showing a relatively high importance index (Figure 5B, Dataset 1D). In total, 35 out of 140 features showed negative importance indexes, yet their effects were negligible (Figure 5B, Dataset 1D), suggesting that these features reduce model performance by introducing potential noise.

Although plasmids can readily acquire new determinants, they can be viewed as groups of linked features, raising a possibility that these features jointly influence the plasmid distribution. We thus wished to investigate the effects of different feature combinations. Consistent with its apparent importance, removal of “smallest” increased the MSE from 0.53 to 0.66 (Figure 5C). We next assessed the impact of dropping two feature columns at the same time; however, no two-feature combinations showed greater effect than removing “smallest”. Facing the fact that we cannot go through all feature combinations (on the order of 10^42^), a random selection identified a combination of seven features which, when removed, increased the MSE from 0.53 to 0.74 (Figure S6A). In this seven-feature combination, only two features, “smallest” and “T2SSE”, had high importance indexes (Figure S6B) and one feature, “large”, had a negative index.

While finding one “bad” seven-feature combination supports our idea that a small subset of features may be important for plasmid distribution, a large dataset would be required to identify features that hold more value for model stack performance. The number of all seven-feature combinations is on the order of 10^11^, precluding testing all of them. Thus, we chose to randomly sample 11,000 seven-feature combinations and selected 33 combinations that have an MSE range of 0.7–0.72, an R^2^ range of 0.17–0.2, and an MSE (*P_sum_* > 1) range of 2.32–2.72, and analyzed feature types therein. The sampled combination pool reassembled the feature types distribution seen in the data matrix, with the mobility type being the most abundant (Figure 5A,D). By contrast, in the 33-combination pool, “plasmid backbone” became the most abundant feature type at the expense of the “mobility”, “transmembrane transporters”, and “unknown” types (Figure 5D).

This result prompted us to compare the plasmid backbone features in all vs. 33 selected seven-feature combinations (Figure 5E). We found that while backbone features were roughly equally distributed among all combinations, “smallest” was present in almost half of the selected combinations, followed by “large”, found in 23% of them (Figure 5E). Another notable difference was a shift to more AT-rich plasmid backbones in the selected set.

## 4. Discussion

In this work, we aimed to identify features that promote the plasmid spread across bacteria. This question is difficult to address because plasmids carry, in addition to genes required for replication, maintenance, and mobilization, an astonishingly diverse collection of “cargo” genes. Those conferring resistance to antimicrobial compounds used in clinical practice are the most infamous, but many other cargo genes may encode beneficial traits only under some circumstances that bacteria encounter in their native habitats. The plasmids in our dataset encode more than 2.5 million predicted proteins, almost half of which cannot be assigned a tentative function (Figure 1C).

Here, we deployed machine learning to identity important features in this complex dataset. After grouping features into broader structural and functional categories, we were left with nearly eight thousand features that did not support good model performance. After the removal of less informative and noise-generating features, a final set of 140 features was used for analysis. Consistent with earlier findings [33], we found that most plasmids were restricted to species level (Figure 3A). Only ~1% of clusters had more than a 70% possibility of multi-phyla distribution (Figure 3A), and most plasmids in these clusters came from one phylum, a result due in part to the strong sampling bias toward Pseudomonadota and Bacillota (Figure 3B and Figure S2). An optimized model stack (Table 1) identified the “smallest” (under 20 kbp) size as the most important feature, either alone (Figure 5B,C) or as part of randomly sampled seven-feature combinations (Figure 5E).

This may appear counterintuitive, because plasmids under 25 kbp would be expected to lack factors necessary for autonomous transfer [7]. Indeed, the mean size in our dataset is 74 kbp and the fraction of conjugative plasmids increases with the plasmid size (Figure 5F). Consistent with earlier estimates [7], the “smallest” group is composed mainly of non-mobilizable and mobilizable plasmids, with just a few conjugative plasmids that may contain a minimal set of transfer genes (Figure 5F). It is notable that the four size groups have a similar fraction of non-mobilizable plasmids (Figure 5F), which can be acquired by a new host through transformation, transduction, or cointegration. These transfer mechanisms favor small-size plasmids [7], the first advantage of being small in spreading.

What other advantages would a small plasmid have in finding a new host? One possibility is that a smaller size could reduce the fitness cost imposed by the synthesis of additional proteins [13], some of which could imperil the host by serving as receptors for phages [52]. However, while some cargo genes may be deleterious, plasmid size per se does not correlate with the fitness cost [53]. The penalty for carrying an additional cargo could be negated by chromosome- or plasmid-encoded nucleoid-associated proteins, which silence the expression of xenogenes [54,55]. Interestingly, we observed a substantial shift toward AT richness among the selected plasmid combinations (Figure 5E), and AT-rich sequences are primary targets for xenogeneic silencing [56].

A second possibility is that small plasmids can evade bacterial defense systems because they have fewer targets for these systems. A recent study of chromosomal and plasmid targets of RM type II systems showed that small plasmids tune their sequences to deplete RM targets, and that plasmids with broader host ranges also show stronger avoidance of RM targets [14]. This strategy is not feasible for large plasmids, which instead encode DNA modification enzymes to inhibit restriction endonucleases [14]; the latter trend may explain enrichment in large plasmids in our selected combinations (Figure 5E).

A third possibility is that being mobilizable may promote the host range expansion. About 60% of <20 kbp plasmids are mobilizable (Figure 5F), and relaxases are the most abundant Pfam hits therein. Conjugal DNA transfer requires two steps mediated by large multi-component protein assemblies [57]. In the first step, following cleavage of the plasmid DNA at oriT and covalent attachment of the DNA end to the relaxase, an integral inner-membrane type-IV coupling protein (T4CP) must connect the relaxase-DNA complex with a type IV secretion system (T4SS) complex. In the second step, the membrane-traversing T4SS, composed of tens of individual protein chains, forms a channel through which the plasmid DNA strand, the relaxase, and other associated proteins [58] are delivered into the recipient cell. The “smallest” plasmids are largely devoid of T4SS genes (Figure 5F) and must rely on a T4SS from a co-resident plasmid for transfer. Furthermore, 94% of plasmids below 30 kbp have been found to lack T4CP [7], making them dependent on a heterologous T4CP for delivery to the T4SS. Since T4CP and T4SS systems coevolve [7], generally limiting DNA transfer to closely-related bacteria, utilization of a foreign T4CP by a relaxase would be expected to enable plasmid transfer to a new host.

We stress that although brevity has the most significant impact on plasmid host range in our analysis, a plasmid’s size alone cannot be used to predict its distribution. The conjugation system, present *in cis* or *in trans*, and replication type are essential for plasmid mobility. Many other factors, including addiction and anti-defense modules, partition systems, and beneficial traits, determine whether the newly acquired plasmid is maintained. As with all such statistical analyses, correlation does not entail causation. However, our study, together with a recent analysis of the plasmid/host arms race [14], highlights the importance of plasmid size and draws our attention to multiple plausible mechanisms by which size could shape plasmid distribution.

Our analysis also revealed other functional features that may contribute to plasmid spread. P-loop NTPases, an ancient group of proteins implicated in diverse cellular processes [59], comprise the second- and third-most important features (Figure 5B). This finding is hardly surprising: the coupling proteins and two separate ATPases of the T4SS transmembrane complex belong to this class [60]. In addition to being parts of the DNA transfer apparatus, P-loop NTPase plays diverse roles in gene expression, cell adhesion, antibiotic efflux, etc. For example, *Escherichia coli* BcsQ (Figure S6) is an essential component of the biosynthesis pathway of cellulose [61], an exopolysaccharide commonly found in *Enterobacteriaceae* biofilms. Cellulose production mediates cell-to-cell adhesion, a decisive factor in efficient conjugation [62]. BcsQ localizes at, and restricts cellulose production to, the cell pole [61], an ideal location for the assembly of the transfer apparatus; TrwB of the *E. coli* R388 conjugative plasmid, the best-studied T4CP, is also targeted to the pole [63].

A rampant spread of multidrug resistance is driven by the conjugal transfer of ARGs, and our analysis shows that β-lactamase has a positive importance index (Figure 5B). β-lactams are the most commonly-used antibiotics [64], and many plasmids in our dataset were obtained from clinical and agricultural samples. Thus, the perceived importance of β-lactamase likely reflects both the acquisition bias and the selection pressure imposed by heavy use of antibiotics in these environments. Importantly, however, our results suggest that the presence of ARGs does not correlate with plasmid distribution—in other words, while plasmid transfer contributes to the rapid dissemination of resistance, this is a one-way street and ARGs do not direct the plasmid traffic.

In our analysis, we have ignored plasmid-encoded proteins whose function cannot be inferred from sequence conservation. These proteins likely provide fitness benefits and counter-defense measures, mediate environmental and cellular signaling, and play other roles. Understanding the function of these proteins and their contribution to bacterial evolution, including the emergence of new threats and benefits to humankind, is an important goal for future studies. A better understanding of plasmid biology will create opportunities for exciting discoveries and may pave the way for harnessing conjugation to manipulate bacterial communities for medicinal or biotechnological purposes [62].

## Figures and Tables

**Table 1:** The optimized hyperparameters of the base models. Only hyperparameters different from the default are shown. The base models involved in the final model stack are highlighted in orange. The LinearRegressor model (options: fit_intercept = false, dropcollinear = false) was used as a meta-learner.

Base Models	Optimized Hyperparameters
RandomForestRegressor	n_trees = 130, n_subfeatures = 70, max_depth = 31, min_samples_split = 2
DecisionTreeRegressor	max_depth = 23, min_samples_split = 4, n_subfeatures = 70, min_samples_leaf = 10
EvoTreeRegressor	loss = :tweedie, nrounds = 70, gamma = 5.1, eta = 0.5, max_depth = 7, colsample = 0.822, nbins = 18
CatBoostRegressor	loss_function = “Quantile”, iterations = 1500, depth = 6, learning_rate = 0.1, l2_leaf_reg = 1, border_count = 10
XGBoostRegressor	eta = 0.2556, gamma = 2.0, max_depth = 5, colsample_bylevel = 0.6, lambda = 5.0
LGBMRegressor	num_iterations = 200, max_depth = 5, feature_fraction = 0.9, learning_rate = 0.055, min_data_in_leaf = 25, lambda_l1 = 2, min_gain_to_split = 1, time_out = 14400

## Data Availability

All plasmid sequences are available from NCBI. The ANI edgeweight file for plasmid clustering analysis, plasmid network files in the Cytoscape format, and the final data matrix for model training and testing have been deposited to DRYAD (https://doi.org/10.5061/dryad.1g1jwsv31, accessed on 3 November 2023). Necessary codes used in this study have been deposited to GitHub (https://github.com/BingWangK/plasmid_project_IAlab, accessed on 27 October 2023).

## Supplementary Materials

The following supporting information can be downloaded at: https://www.mdpi.com/article/10.3390/genes14112044/s1, Figure S1: Illustration of the sum of distribution possibility (*P_sum_*) calculation. Please also see the corresponding METHOD part; Figure S2: Host taxonomic lineage distribution on phylum level in our dataset. The family distributions of Bacillota and Pseudomonadota are also shown; Figure S3: Resolution scan for plasmid clustering by using the Leiden algorithm with the constant Potts model. The best resolution is indicated as a blue dot, and the values are (resolution, modularity); Figure S4: Correlation of ARGs/plasmid sizes and *P_sum_*. *P_sum_* is shown as the blue area, and features are orange scatters. The Pearson correlation coefficient (r) between *P_sum_* and the tested features was calculated in Excel. (A) For each cluster, the fraction of plasmids containing ARGs was calculated. Then, the Pearson correlation was calculated. (B, C, D, and E) show the correlation result of the features “smallest”, “small”, “average”, and “large”, respectively; Figure S5: Performance of the optimized base models on testing datasets. The dataset was split randomly into training and testing datasets 100 times. The model stack was trained on the training dataset and tested on paired unseen testing datasets. MSE, mean squared error. MSE (*P_sum_* > 1), MSE for the clusters with *P_sum_* > 1. Data are shown as mean ± SD (*n* = 100); Figure S6: The effect of the “bad” 7-feature combinations. (A) Model stack performance on testing dataset after removal of 7 features. Values are reported as mean ± SD (*n* = 100) calculated as (Figure 4). (B) The annotation of 7 features used in panel (A). The functions for the Pfam models were inferred from InterPro (https://www.ebi.ac.uk/interpro/, accessed on 1 November 2023); Dataset 1, datasets generated in this study. (A) Plasmid included in the final 1125 clusters. Metadata of plasmids included in the final 1125 clusters. Plasmid NCBI ID, the assigned clusters, plasmid name, genome assembly ID, plasmid host taxonomic lineage, plasmid size, GC content, and topology are present. The predicted mobilities by Mob-typer are shown. (B) Plasmids that were excluded from the final dataset. The removing reasons are indicated. (C) Distribution possibility of each cluster. (D) The importance index of the 140 features in the final data matrix. The 12 feature types are indicated. MSE_test, mean squared error on testing dataset; MSE_test_std, the standard deviation of MSE_test; R2_test, coefficient of determination on testing dataset; R2_test_std, the standard deviation of R2_test; MSE_test (P > 1), MSE for those clusters with *P_sum_* > 1; MSE_test (P > 1)_std, standard deviation of MSE_test (P > 1); MSE_test absolute differences, MSE_test of a tested feature minus the MSE_test of baseline; Importance index, the MSE_test absolute differences divided by 0.124068. (E) Plasmids containing ARGs and the assigned clusters.
