# Supplementary figures and images for: Machine Learning Suggests That Small Size Helps Broaden Plasmid Host Range
Source: Genes (Basel). 2023 Nov 5;14(11):2044. doi: 10.3390/genes14112044 (PMC10670969; doi:10.3390/genes14112044)

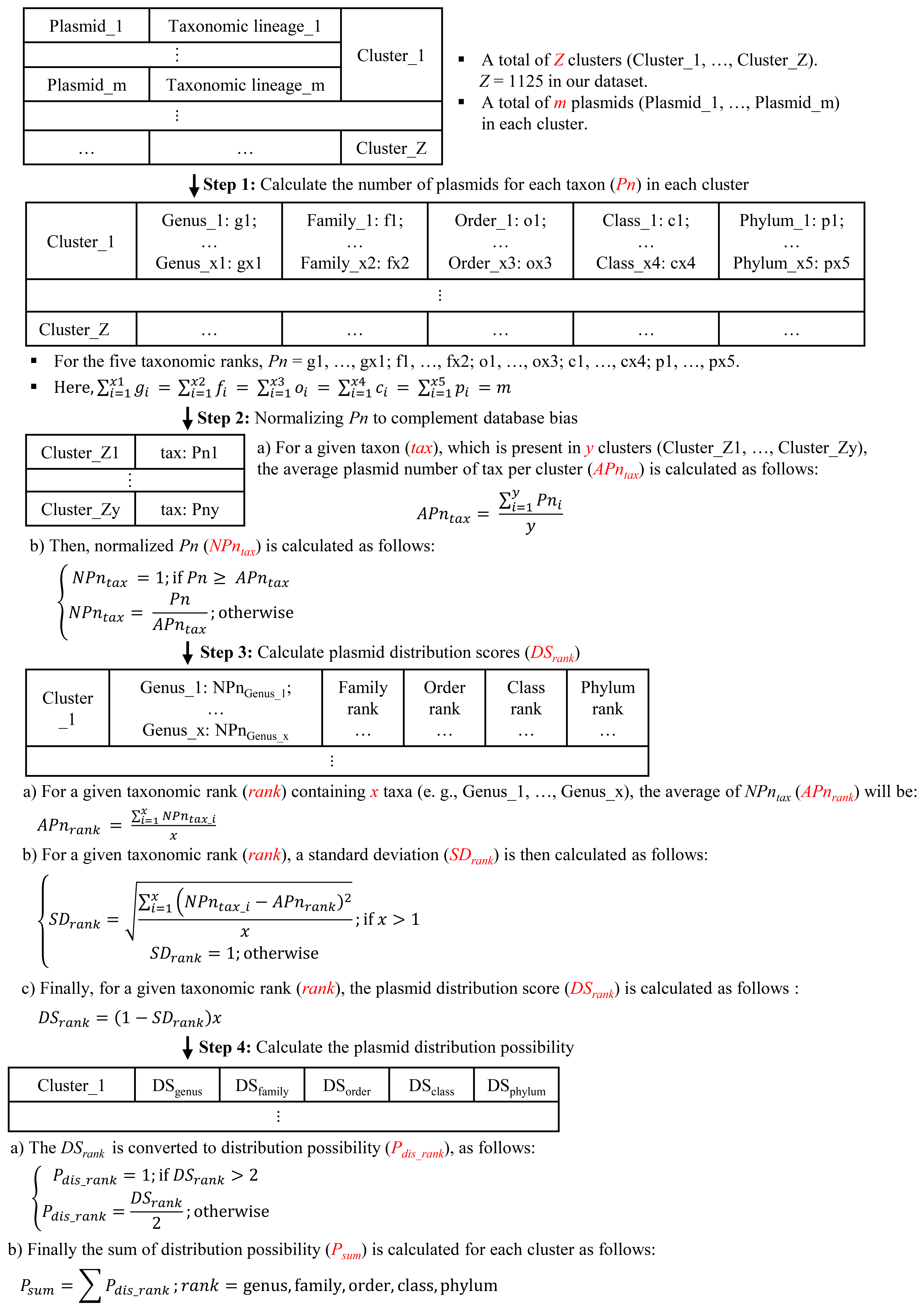

Supplement: Supplementary file 1 [file genes-14-02044-s001.zip › genes-2691684-supplementary/Figure S1.tif]

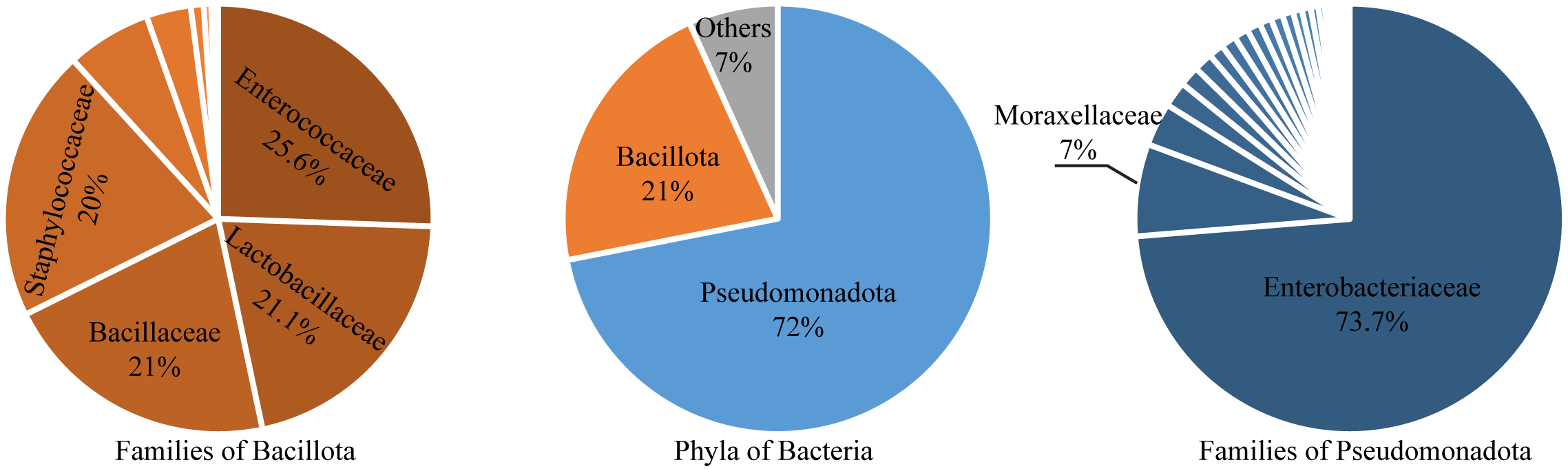

Supplement: Supplementary file 1 [file genes-14-02044-s001.zip › genes-2691684-supplementary/Figure S2.tif]

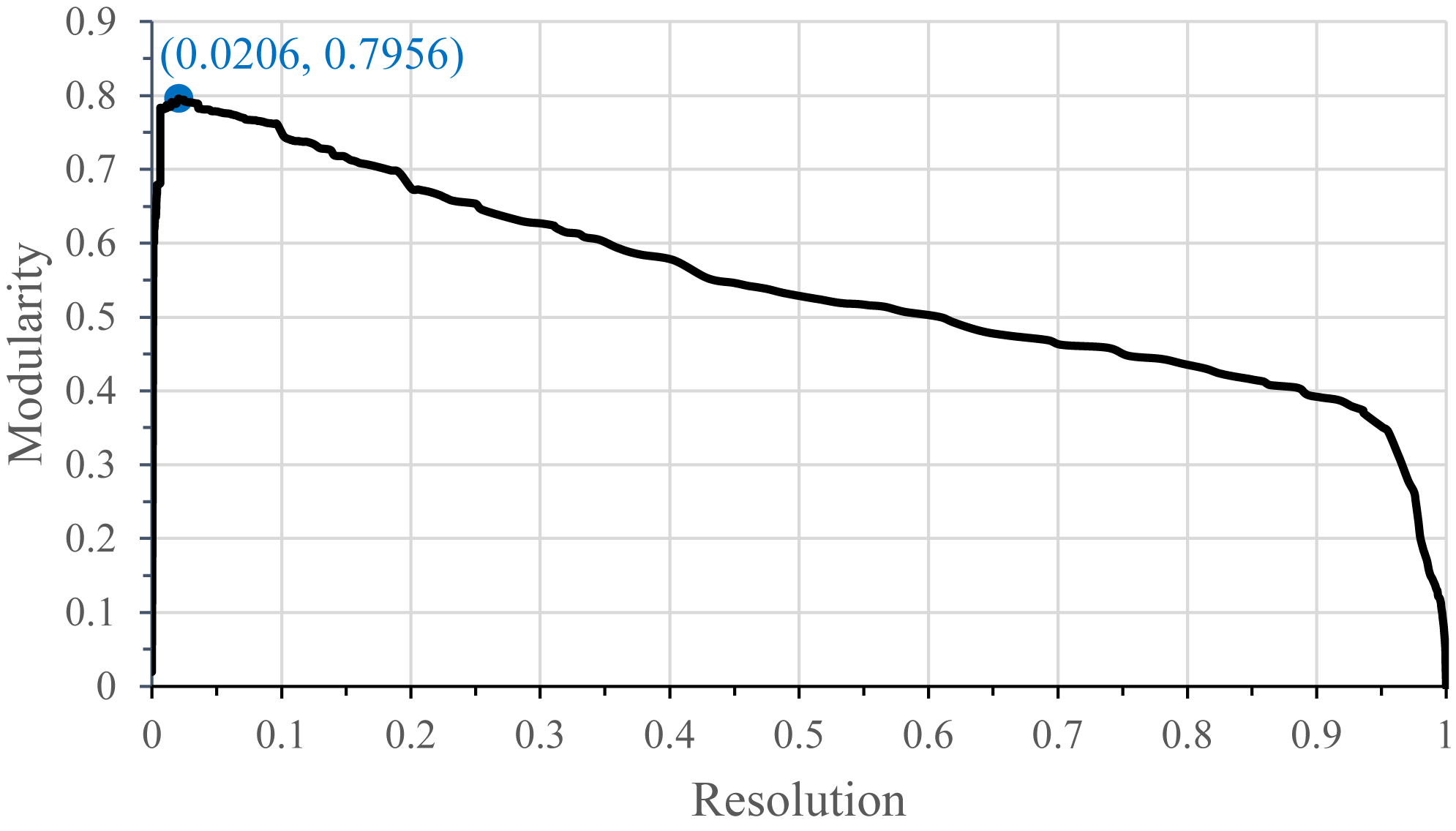

Supplement: Supplementary file 1 [file genes-14-02044-s001.zip › genes-2691684-supplementary/Figure S3.tif]

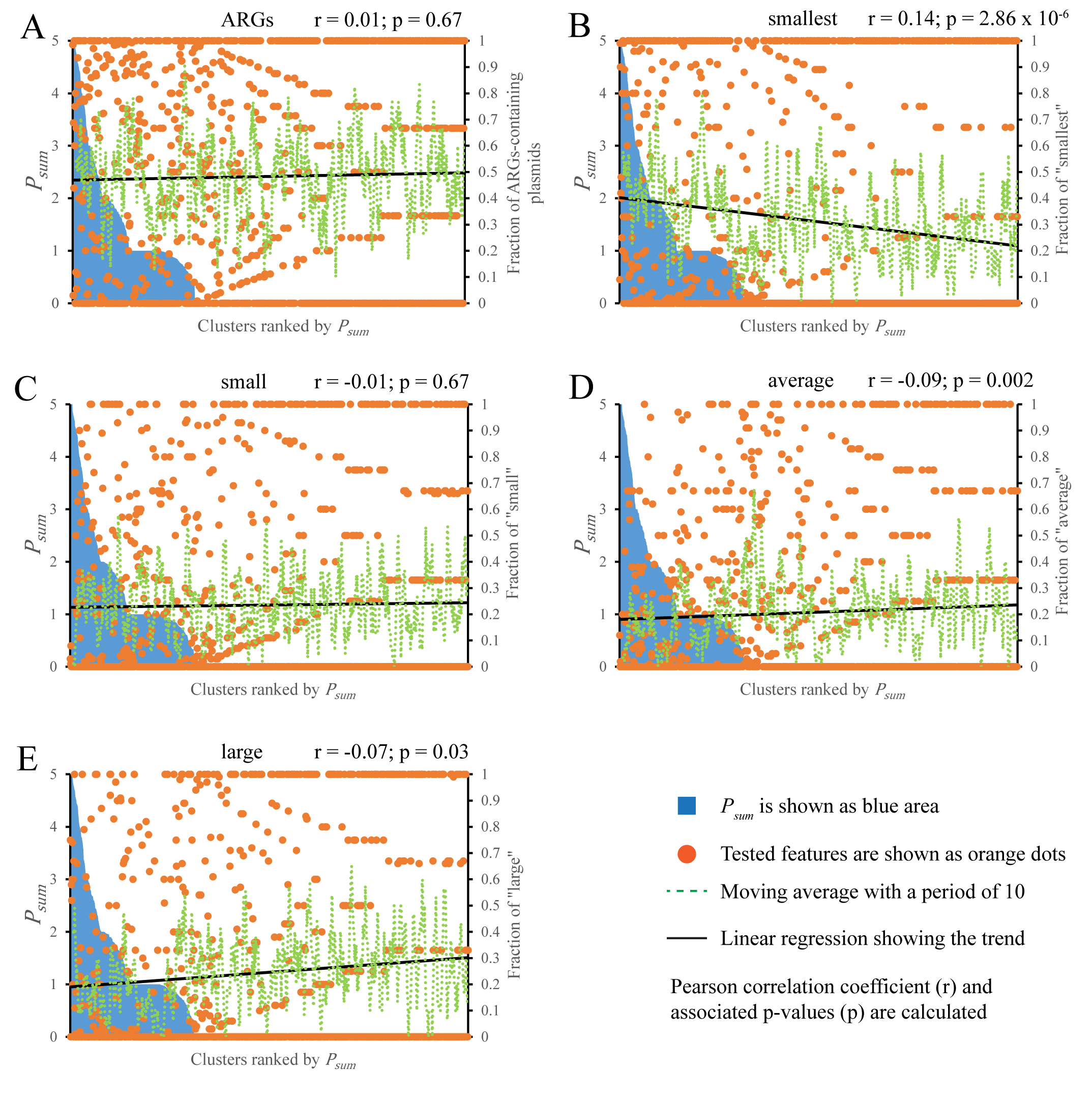

Supplement: Supplementary file 1 [file genes-14-02044-s001.zip › genes-2691684-supplementary/Figure S4.tif]

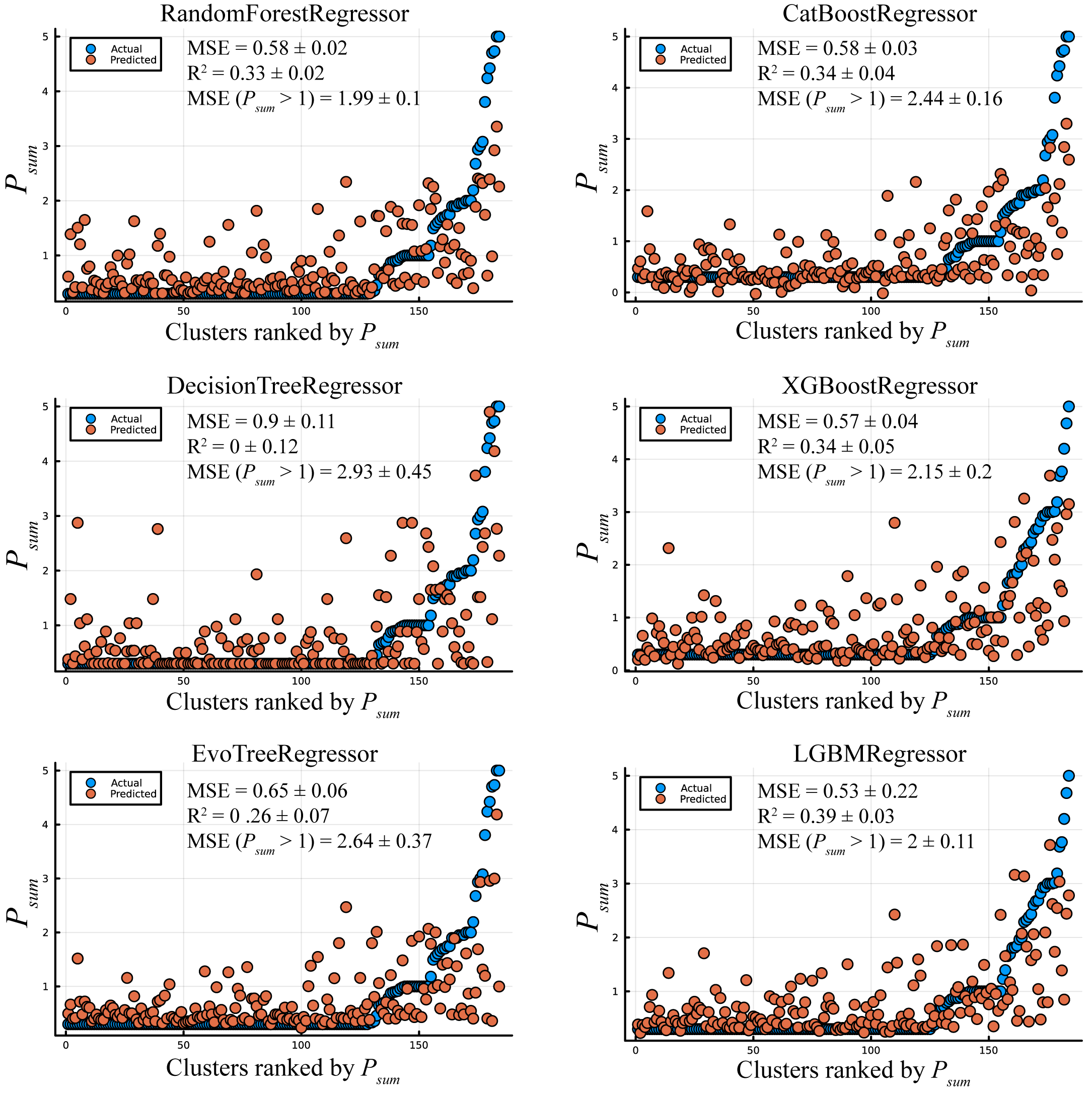

Supplement: Supplementary file 1 [file genes-14-02044-s001.zip › genes-2691684-supplementary/Figure S5.tif]

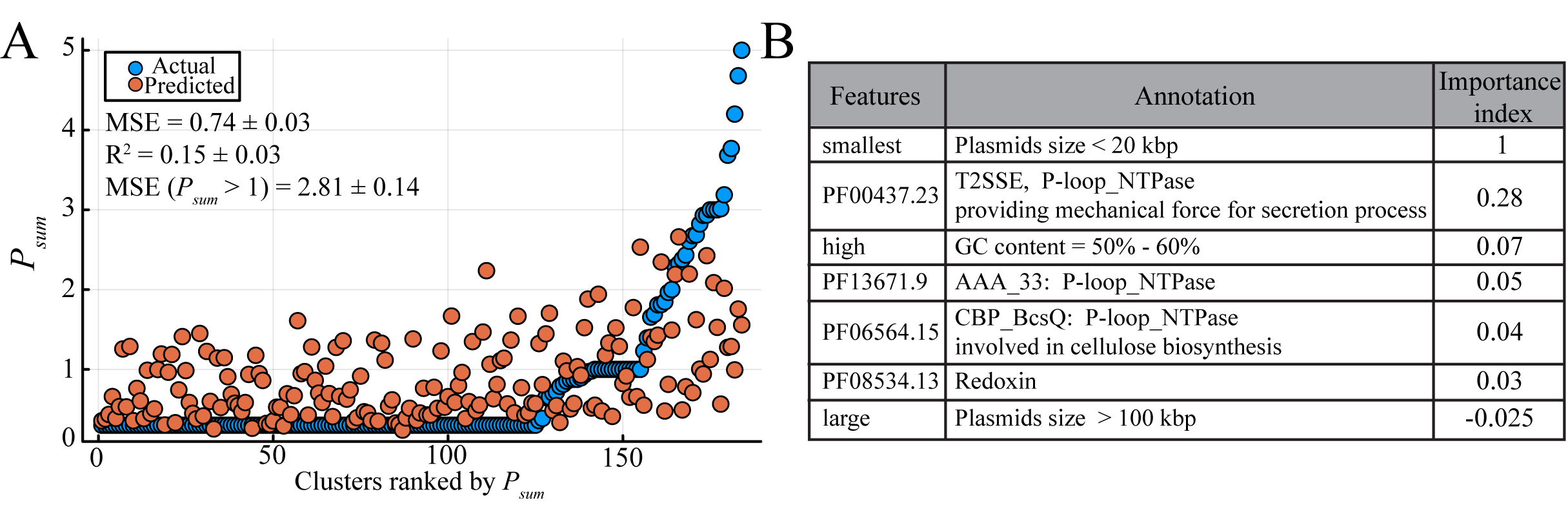

Supplement: Supplementary file 1 [file genes-14-02044-s001.zip › genes-2691684-supplementary/Figure S6.tif]
